# Intestinal Regulatory T Cells as Specialized Tissue-Restricted Immune Cells in Intestinal Immune Homeostasis and Disease

**DOI:** 10.3389/fimmu.2021.716499

**Published:** 2021-08-04

**Authors:** Justin Jacobse, Jing Li, Edmond H. H. M. Rings, Janneke N. Samsom, Jeremy A. Goettel

**Affiliations:** ^1^Department of Pediatrics, Willem-Alexander Children’s Hospital, Leiden University Medical Center, Leiden, Netherlands; ^2^Department of Pathology, Microbiology, and Immunology, Vanderbilt University, Nashville, TN, United States; ^3^Department of Medicine, Division of Gastroenterology, Hepatology and Nutrition, Vanderbilt University Medical Center, Nashville, TN, United States; ^4^Department of Pediatrics, Sophia Children’s Hospital, Erasmus University, Erasmus University Medical Center, Rotterdam, Netherlands; ^5^Laboratory of Pediatrics, Division of Gastroenterology and Nutrition, Erasmus University Medical Center, Rotterdam, Netherlands; ^6^Program in Cancer Biology, Vanderbilt University School of Medicine, Nashville, TN, United States; ^7^Vanderbilt Institute for Infection, Immunology, and Inflammation, Vanderbilt University Medical Center, Nashville, TN, United States; ^8^Center for Mucosal Inflammation and Cancer, Vanderbilt University Medical Center, Nashville, TN, United States

**Keywords:** intestine, regulatory T (Treg) cells, homeostasis, IBD, intestinal inflammation

## Abstract

FOXP3^+^ regulatory T cells (Treg cells) are a specialized population of CD4^+^ T cells that restrict immune activation and are essential to prevent systemic autoimmunity. In the intestine, the major function of Treg cells is to regulate inflammation as shown by a wide array of mechanistic studies in mice. While Treg cells originating from the thymus can home to the intestine, the majority of Treg cells residing in the intestine are induced from FOXP3^neg^ conventional CD4^+^ T cells to elicit tolerogenic responses to microbiota and food antigens. This process largely takes place in the gut draining lymph nodes *via* interaction with antigen-presenting cells that convert circulating naïve T cells into Treg cells. Notably, dysregulation of Treg cells leads to a number of chronic inflammatory disorders, including inflammatory bowel disease. Thus, understanding intestinal Treg cell biology in settings of inflammation and homeostasis has the potential to improve therapeutic options for patients with inflammatory bowel disease. Here, the induction, maintenance, trafficking, and function of intestinal Treg cells is reviewed in the context of intestinal inflammation and inflammatory bowel disease. In this review we propose intestinal Treg cells do not compose fixed Treg cell subsets, but rather (like T helper cells), are plastic and can adopt different programs depending on microenvironmental cues.

## Introduction

The immune system provides host protection against pathogenic microorganisms as well as anti-tumor immunity. Importantly, regulatory mechanisms prevent immune reactivity against self- and harmless foreign antigens. However, in some individuals this form of tolerance is compromised leading to various autoimmune conditions. Key observations made in the 1960’s and 1970’s began to unveil the mediators of autoimmunity and the concept of immunological tolerance. In the early 60’s, Jacques Miller showed that thymectomy of mice at birth results in a lack of peripheral T cell responses to skin grafts, identifying the thymus as the essential organ of T cell development (1, 2). Yet, it was the seminal finding in 1970 by Richard Gershon that the thymus also gives rise to cells essential for immune tolerance (3). Then in 1976, the developmental timing of tolerance was first reported by Kojima and colleagues who showed that thymectomy in mice between 2-4 days after birth leads to autoimmune thyroiditis, whereas no lesions are observed if the thymectomy is performed at birth or after day 5 (4). This autoimmunity following thymectomy can be prevented by the adoptive transfer of thymocytes or splenocytes from adult euthymic mice (4–6). Intriguingly, in the thymus a limited number of autoreactive CD4^+^ T cells differentiate into highly suppressive T cells, termed regulatory T cells (Treg cells), in a process known as agonist selection to ensure central tolerance to self-antigens, and thereby prevent autoimmunity (7–9). Despite the recognition that T cells could play a “suppressor” role in the control of effector T cell responses, it would take nearly two decades to define this population. Seminal work by Fiona Powrie in 1993 described two populations of T cells that can induce or protect immunodeficient mice from chronic colitis following adoptive transfer of T cells based on the expression level of CD45RB (naïve T cells are CD45B^high^) (10). Shortly after, Sakaguchi et al. reported that CD4^+^ T cells that immunosuppress T helper cells express the cell surface marker CD25 (11) and that these cells reside within the CD45RB^low^ fraction of T cells, the same fraction that prevents colitis mediated by adoptive transfer of CD45RB^high^ T cells (12).

Treg cells are essential for maintaining both central as well as peripheral immunological tolerance (13) and are found in many tissues and mucosal surfaces including the intestine. The intestinal mucosa is lined by a single cell layer of columnar epithelial cells with the lamina propria (LP) below the epithelial cells and muscularis mucosa at the base. The LP serves as an effector site, whereas adaptive immune cells are primed within inductive sites including lymph nodes that drain anatomically distinct regions of the intestine and/or mucosa-associated lymphoid tissue such as Peyer’s patches. For example, the duodenum drains into a lymph node embedded in pancreatic tissue, while the jejunum, ileum, cecum, and ascending colon drain into the mesenteric lymph nodes (mLNs). Two small lymph nodes in the pancreatic tissue also drain the transverse colon and descending colon while the distal colon and rectum primarily drain to caudal lymph nodes (14–19).

Just as different anatomical regions of the intestine have specific functions, immune cells residing in, or trafficking to, distinct regions of the gut carry out specialized functions. Naïve T cells circulate from blood into lymph nodes *via* afferent lymphatic vessels where they interact with antigen-presenting cells (APCs) such as dendritic cells (DCs) that have acquired peptides in the intestinal lumen or LP and migrated to the draining lymph nodes. Together, the local microenvironment in the lymph nodes draining these specific intestinal segments and the function of the resident DCs contribute to mounting either tolerogenic or pro-inflammatory adaptive immune responses. Besides the draining lymph node compartment, the intestinal microenvironment in the various segments of the GI-tract is also markedly different. In the small intestine (SI), the relative amount of soluble, luminal food antigens decreases from the duodenum to the terminal ileum and colon. In stark contrast, the large intestinal micro-environment is characterized by high abundance and diversity of commensal bacteria.

One of the major functions of the intestinal immune system is to maintain immune homeostasis among the non-pathogenic gut microbiome, harmless exogenous food antigens, pathogenic microbes, and damage-associated self-antigens. Microenvironmental cues by both immune and non-immune cells alike help define the phenotype and function of intestinal Treg cells. Suppression of effector T cells by Treg cells requires a T-cell receptor (TCR)-dependent signal. Under physiological conditions, Treg cells regulate immune responses to self-antigens, microbes, and food antigens. While Treg cell activation requires a TCR-specific signal, Treg cells can also suppress effector T cells with different antigen specificity through a process called bystander suppression that contributes to overall immune homeostasis. In addition, Treg cells can secrete immunosuppressive cytokines such as interleukin (IL)-10. Consequently, loss of suppressive function caused by defective Treg cells results in intestinal inflammation in mice and contributes to inflammatory bowel disease (IBD) in humans.

This review will provide the reader with the current evidence regarding the role Treg cells play in maintaining immune homeostasis in the colon and SI, both in mouse models and in human disease.

## Intestinal Treg Cells Are a Diverse Class of Immunoregulatory Immune Cells

The most commonly studied Treg cell populations are defined by expression of CD4, CD25, and Forkhead box P3 (FOXP3). Expression of the transcription factor FOXP3 is necessary to bestow suppressive capacity on Treg cells and maintain the Treg cell phenotype [Reviewed in (20)]. Unfortunately, classification of human FOXP3^+^ cells is not as definitive as in mice as both FOXP3 and CD25 can be observed in activated conventional T cells (21). To identify human Treg cells by flow cytometry, a minimum set of cell surface markers consisting of CD3, CD4, CD25, CD127, and FOXP3 are used with Ki67 and CD45RA providing additional information about Treg cell proliferation and activation status (21, 22). Studying human Treg cells is further complicated by FOXP3 being an intranuclear protein, and as such requires fixation and permeabilization that limits the ability to purify cells using cell sorting for functional characterization.

Other regulatory CD4^+^ T cells have been described that do not express FOXP3 but nevertheless regulate intestinal immune responses, possessing both distinct and similar features of FOXP3^+^ Treg cells. Type 1 regulatory T cells (Tr1) were initially described by Roncarolo et al. (23, 24) as CD4^+^FOXP3^neg^ T cells that co-express CD49b, LAG-3, CD226, CCR5, and PD1 (25, 26). In contrast to FOXP3^+^ Treg cells, Tr1 cells are more abundant in the SI and Peyer’s patches (PP) and rapidly secrete IL-10 and TGFβ upon stimulation in the absence of IL-4 (27). Functionally, Tr1 cells suppress antigen-specific T-cell proliferation in an IL-10-dependent manner and are protective in the adoptive naïve T cell transfer model of colitis (23, 24, 28). Whereas both FOXP3^+^ Treg cells and Tr1 cells produce IL-10, Tr1 cells seem to be especially important for maintaining tolerance to commensal bacteria. Nevertheless, FOXP3^+^ Treg cells are still necessary for initial tolerance induction (29–31). For the remainder of this review we will focus on FOXP3^+^ Treg cells.

According to the recommended nomenclature, Treg cells can be subdivided into tTreg cells when they originate from the thymus and pTreg cells when they are locally induced. In addition, naïve T cells can be cultured *in vitro* under conditions that promote Treg cell induction; these are commonly referred to as iTreg cells (32). *In vitro* induced Treg cells differ from *ex vivo* Treg cells (33) and this should be taken into consideration when interpreting studies performed with iTreg cells. Regardless of their site of origin or ontogeny, Treg cells must exhibit suppressive properties to be considered *bona fide*. Assessing Treg cell function is generally carried out using *in vitro* suppression assays. Despite some limitations (34), this assay quantifies the proliferation of conventional T cells when activated in the presence of Treg cells. Altogether, Treg cells are defined by a collection of markers and confirmation of suppressive function commonly performed *via in vitro* suppression assay.

The peripheral Treg cell pool can be further classified into three groups based on location and function: central, effector, and tissue-resident. The main properties of these populations, as reviewed in detail by Liston and Gray (35), will be summarized here. Central Treg cells (mouse- CD62L^high^CCR7^+^ or CD45RA^high^CD25^low^) are considered to be naïve and represent the majority of Treg cells in circulation and in secondary lymphoid organs. Effector memory, or activated Treg cells, (mouse- CD62L^low^CCR7^low^CD44^hi^KLRG1^+^CD103^+^ or CD25RA^low^CD25^hi^) possess characteristics of conventional activated CD4^+^ T cells with recent antigen exposure and are less abundant. In contrast to the central and effector Treg cells, tissue Treg cells reside in non-lymphoid tissue such as the colon. In absence of inflammation, the majority of Treg cells in the gut are tissue-resident pTreg cells.

Like conventional T cells, Treg cells are governed by transcription factors (e.g., T-BET, STAT3, and IRF4) that also regulate the function of effector T cells during Th1-, Th2-, and Th17-mediated inflammation respectively (36–38). Thus, it is not surprising that the transcriptome of tissue Treg cells varies according to their location which regulates expression of these transcription factors [Reviewed in (39)]. For many Treg cell genes regulated by the local environment, expression tends to reflect a gradient rather than binary, suggesting plasticity and adaptation to their microenvironment (40). Although not entirely definitive, some markers used to discriminate intestinal Treg cells from each other are RORγt (microbiome, highly suppressive), IL-33R (tissue protection during inflammation), IL-10 (suppression of autoimmunity), GATA3 (suppression of Th2), HELIOS (probable thymic origin), and NRP1 (probable thymic origin) (**Table 1**).

**Table 1:** Markers of intestinal Treg cells and the gist of their phenotype translated to intestinal immune homeostasis.

Marker	Induction	Function
IL-10	Induced by microbiome (41).	Regulates Th1 response (42, 43).
	Requires innate immune cells (44).	
	Requires BLIMP-1 for function (45).	Controls Th17-type inflammation in the colon *via* STAT3 (46–48).
Rorγt	Induced in the colon by microbiome (49).	Controls Th1-type and Th-17 type inflammation (50).
	To lesser extent, may be induced by dietary antigens in absence of microbiome (51).	
IL-1R		Characteristic of unstable Treg cells (52, 53).
IL-33R		Associated with tissue repair (54).
		Characteristic of stable Treg cells (52).
IL-23R		Inhibits IL-33 responsiveness (54).
		Function in intestine unknown.
GATA3	Decreased in absence of CNS1 (55).	Controls spontaneous Th2-type enteritis (55).
	Induced by high affinity TCR ligands (55).	GATA3-deficient Treg cells produce more IL17A (56).
HELIOS	Supported by IL-33 (50).	Proposed marker for tTreg cells (57).
NRP1	Expressed during inflammation (58).	Proposed marker for tTreg cells (58, 59).
		Promotes Treg cell activity (60, 61).

## The T Cell Receptor Repertoire of Intestinal Treg Cells Is Influenced by Luminal Antigens

In the intestine, Treg cells have a distinct TCR repertoire (repertoire) from both non-Treg cells as well as Treg cells residing in other organs, suggesting that tissue-specific factors shape the Treg cell repertoire (62). Germ-free (GF) mice have fewer intestinal Treg cells than conventionally housed mice. When CD4^+^CD25^+^ Treg cells are isolated from conventionally housed mice or GF mice and co-injected with naïve T cells in the adoptive T cell transfer model of colitis, recipients of GF Treg cells exhibit increased inflammation (63). It has been suggested that these effects may be due to the limited repertoire of GF Treg cells as compared to the larger repertoire of Treg cells isolated from conventionally housed mice (64). This would be consistent with locally induced intestinal Treg cells having TCRs specific for microbial antigens and small quantities of antigens being sufficient for efficient pTreg induction (65). Indeed, *in vitro* data show that some TCRs of colonic Treg cells are specific for food or microbial antigens in stool from conventional mice but not from GF mice (62). While a limited Treg cell repertoire is one possible explanation, it has also been shown that CD4^+^CD25^+^ cells from GF mice express less *Foxp3* and exhibit reduced functionality, which could also account for the increased inflammation (66). Interestingly, a study from the Rudensky laboratory showed that activated CD25^+^ cells in *Foxp3* ^-/-^mice have a similar repertoire as Treg cells in wild-type mice, which implies a cell-intrinsic mechanism directing Treg cell TCR development but likely restricted to those of thymic origin (67). Furthermore, mice that develop spontaneous colitis due to defects in IL-2, IL-10, or TGFβ have many effector and memory T cells with TCRs that in wild-type mice are found on colonic FOXP3^+^ cells (62). These studies suggest that Treg cells in physiological conditions exert TCR-specific suppression of inflammation mediated by other T cell subsets. The importance of TCR diversity in Treg cells comes from a study showing that Treg cells isolated from mice with a limited repertoire are not able to suppress the development of spontaneous, microbiome-dependent, Th17-type intestinal inflammation (68).

It is clear that a proportion of colonic Treg cells exhibit specificity for colonic luminal antigens, mostly microbial (62, 69). However, the ontogeny of intestinal Treg cells with TCR specificity for the gut microbiota is not entirely known. The TCR specificity of a Treg in itself does not necessarily pre-determine the location in which the cell will reside. This follows from the observation that a T cell expressing a TCR from a pTreg becomes either a pTreg cell or an intraepithelial lymphocyte, dependent on the location (70). T cells in this model were generated by transferring the nucleus of an Nrp1^low^Foxp3^+^ cell from the mLN to the donor cell, i.e. generating a transnuclear mouse. In the SI these cells are poised to become intraepithelial lymphocytes (in a FOXP3-independent manner), whereas in the mLN they are poised to become pTreg cells, as evidenced by accumulation of these cells in these respective locations (70).

Although colonic Treg cell TCRs retrovirally expressed in thymocytes were reported to be unable to facilitate thymic Treg cell differentiation (62), a fraction of thymic Treg cells share TCRs with pTreg cells indicating that a portion of intestinal Treg cells originate from the thymus (69). Surprisingly, a recent landmark paper from the Diehl laboratory shows that microbial antigens are presented to T cells in the thymus, suggesting that a part of the intestinal Treg cells *can* originate from the thymus (71). Additional evidence highlighting the influence of the microbiome on colonic Treg cell TCR comes from Cebula et al. who reported that the repertoire of colonic Treg cells changes following antibiotic treatment (69). In this study, antibiotic treatment decreased some TCR clones and expanded other TCR clones. However, overall, antibiotic treatment did not alter the repertoire diversity of colonic Treg cells as compared to other Treg cells. Furthermore, the TCR clones that did change with antibiotic treatment were also found on thymic Treg cells. Finally, colonic Treg cells TCRs cloned into hybridomas were reactive against fecal extracts were also found on thymic Treg cells. These findings can be interpreted to mean at least some intestinal Treg cells originate from the thymus (69).

Collectively, these highly informative studies raise new questions that need to be reviewed with a few considerations. Several of the studies listed above employ fixed TCR-β chains. When using a transgenic mouse with fixed TCR-β, the TCR diversity and antigen specificity is determined by the TCR-α chain, allowing for analysis of the diversity of the repertoire in different T cell subsets with high certainty but not reflecting the true diversity of the human repertoire (72). Nevertheless, as a whole the data strongly argue that a large proportion of intestinal Treg cells have TCRs specific to microbial antigens.

## Mucosal CD103^+^ Dendritic Cells and Retinoic Acid Are Pivotal Players in the Induction of Small Intestinal Treg Cells

**Figure 1:**
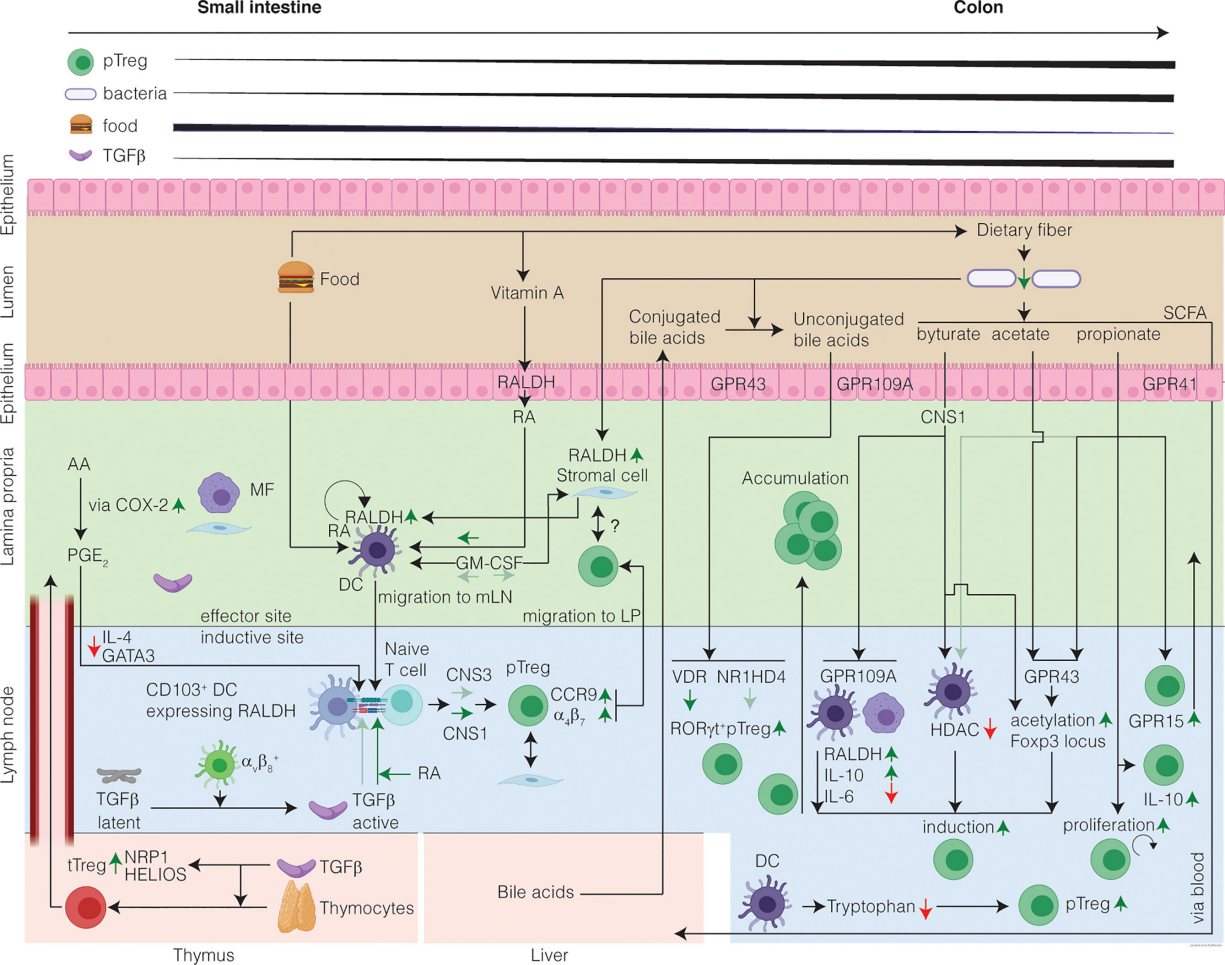
**The healthy intestine favors Treg-cell induction in response to harmless antigen and local microenvironmental conditioning maintains the Treg cell phenotype.** The mechanisms of intestinal Treg cell induction depend on the structure of the encountered antigen and on the inductive site: in the SI encounter of harmless food antigens predominates eliciting DC-mediated Treg cell induction from naïve T cells. In the colon, commensal microbiota produce SCFA which in presence of TGFβ induce RORγt^+^ Treg cells. In the SI inductive sites, DC-derived TGFβ induction converts circulating naïve T cells into pTreg cells, which is further potentiated by diet-derived RA. Treg cell induction in the SI is CNS1-dependent with a minor contribution from CNS3. In the colon inductive sites, the SCFA induce Treg cells. SCFA induce acetylation of the Foxp3 locus and alter the phenotype of the pTreg cells by increasing RORγt. Moreover, bile acids unconjugated by the microbiome contribute to the induction of colonic RORγt^+^ Treg cells. After induction, pTreg cells migrate from the inductive sites to the LP, where they accumulate and maintain immune homeostasis. In the LP, Treg cells might interact with stromal cells, which receive signals from the microbiome, through a cell-contact dependent mechanism. A portion of the intestinal Treg cells is tTreg cells, induced in the thymus in presence of TGFβ, and that have migrated to the intestine and are highly expressing putative markers for tTreg cells. Finally, a gradient of Treg cells exists increasing from SI to colon. Question marks indicate research areas that remain relatively unexplored.

Oral tolerance develops upon first encounter of the antigen ovalbumin (OVA), which is a harmless exogenous chicken egg white protein and the most widely used model food antigen. Oral tolerance to OVA is critically dependent on resident intestinal DCs carrying the antigen to gut-associated lymphoid tissue (GALT) and mLN (73). In contrast to the spleen, OVA-specific FOXP3^+^ Treg cells are preferentially induced in these two sites, emphasizing the compartmentalization of this response (**Figure 1**) (74–76). Within 24-48 hours after OVA oral gavage, CD25^+^ Treg cells differentiate in PP and mLN and exhibit functional suppressive capacity, conferring oral tolerance when adoptively transferred to OVA-naïve recipient mice (75). Subsequent studies demonstrated that a large proportion of these mucosally-induced pTreg cells express FOXP3 (74, 76). Since Treg cell induction in response to a harmless dietary antigen occurs with higher frequency in mucosal *versus* non-mucosal draining lymphoid tissue, it was questioned whether particular subsets of DCs in the PP and mLN are required. As compared to splenic DCs, SI DCs are better able to convert naïve CD4^+^ cells to stable suppressive FOXP3^+^ cells when stimulated with anti-CD3 in the presence of TGFβ *in vitro*. Furthermore, DCs in the SI and cLP, as well as their respective draining lymph nodes, can be divided into four subsets based on CD11b and CD103 expression (19). CD103^+^ DCs are generally CD11c^+^MHCII^+78^ and represent a significant population of SI DCs, with a smaller population of cLP DCs being CD11c^+^CD103^+^. SI LP CD103^+^ DCs are more efficient in inducing Treg cells as compared to LP CD103^neg^ DCs, which is attributed to retinoic acid (RA) being a co-factor for TGFβ-dependent Treg cell induction, with TGFβ thought to be the rate-limiting factor (74, 76, 77).

The SI microenvironment is high in RA as enterocytes metabolize retinol (vitamin A) to RA *via* retinal aldehyde dehydrogenases (RALDH) (78). CD103^+^ DCs in the mLN also highly express RALDH (encoded by *Aldh1a2)* and produce RA, whereas CD103^neg^ DCs in the mLN do not (76). In addition, TGFβ induces CD103^+^ expression on DCs (77) and selectively potentiates the ability of CD103^+^ but not CD103^neg^ DCs to induce Treg cells in a dose-dependent manner. Moreover, RA enhances active TGFβ to induce FOXP3 in T cells *in vitro* by both GALT and splenic DCs, but this is specific to the CD103^+^ DC subset (74, 76). Furthermore, IL-2 is required for this induction as anti-IL-2 abolishes *in vitro* induction of FOXP3^+^ cells in the presence of RA and TGFβ. Another contributing factor to DC-mediated Treg-cell induction is the interaction of mucosal DCs with stromal cells, which has been shown to imprint RA production on DCs. Stromal cell production of RA is independent of vitamin A, but rather depends on the microbiome (79). Importantly, these DC-mediated induced Treg cells are functional *in vivo* as demonstrated by their ability to suppress colitis in the adoptive T cell transfer setting (77). Altogether, these studies show that a specialized subset of CD103^+^ DCs present in GALT favors the Treg cell induction *in vitro* and *in vivo*, requires TGFβ and IL-2, and is potentiated by RA. The exact mechanism by which RA potentiates TGFβ to induce Treg cells remains to be elucidated.

Like Treg cells, the distribution of DCs along the GI-tract is not random. The fraction of CD11b^+^CD103^+^ DCs in the colon is lower than in the SI, whereas the CD11b^neg^CD103^+^ DC subset comprises a larger fraction of the cLP DCs, with a similar pattern in their respective draining lymph nodes (19). Consistent with this gradient and the above described function of CD103, RALDH activity in the SI is highest as compared to other compartments such as the mLN and colon (80). Additionally, CD103 expression by DCs in the mucosa is higher than DCs in the gut draining lymph nodes (19). Interestingly, only SI DCs, but not cLP DCs, can present OVA in the mLN (19). Despite this gradient of CD11b^+^CD103b^+^ DCs, Treg cell induction to soluble protein antigen delivered *via* rectal enema can be established in the colon draining iliac/caudal lymph nodes (ILN) independent of CD103^+^ DCs (18). Of note, it has recently been shown that induction of CD11b^+^CD103^+^ DCs is dependent on TGFβ signaling (81), and it is suspected that TGFβ, which is equally present at the mRNA level in the ILNs and mLN, may strongly contribute to DC-mediated FOXP3^+^ Treg cell induction in both ILN and mLN. In keeping with this pivotal role of TGFβ, SI CD103^+^ DCs can induce FOXP3^+^ Treg cells independently of RA *via* integrin α_v_β_8_-mediated activation of TGFβ (82, 83). Collectively, these data highlight the interplay between resident mucosal DCs, RA, and TGFβ, and the local microenvironment in the mucosa draining lymphoid tissues are essential for the induction of SI Treg cells to promote immunological tolerance.

## Stromal- and Epithelial-Dependent Induction of Intestinal Treg Cells

The contribution of non-immune cells to intestinal immune regulation has not been well-characterized. In humans, direct interactions between intestinal epithelial cells and the immune system contribute to intestinal Treg cell development *via* DCs. This is especially relevant in patients with Crohn’s disease, where epithelial cells have been shown to be functionally altered (84). Epithelial cells can induce tolerogenic DCs directed by TGFβ and RA (84) as well as produce thymic stromal lymphopoietin, which is also known to contribute to Treg cell induction (84, 85). Thus, intestinal epithelial cells are important players in Treg cell induction and function.

Directly underlying the intestinal epithelium, stromal cells interact with both immune and epithelial cells and have been proposed to regulate immune responses (86, 87). Small intestinal stromal cells induce DCs to produce RA under influence of the microbiome and increase iTreg cell differentiation (79). Additionally, stromal cells from the mLN are essential to induce CCR9 and α4β7 on T cells in a RALDH-dependent manner (88), and neonatally imprinted mLN stromal cells are also required for the Treg cell induction (89, 90). These stromal cells from the mLN can also produce vesicles that carry TGFβ and can induce Treg cells (91). Kashiwakura et al. showed that stromal cells from the spleen and lymph nodes have cell-to-cell contact with Treg cells from these same tissues to decrease apoptosis in a CD2-dependent manner (92). An elegant study showed that human Treg cells can be induced from peripheral blood naïve T cells in the presence of colonic stromal cells in a PGE_2_-dependent manner. When these stromal cells are derived from patients with IBD, their capacity to induce Treg cells is slightly reduced (93). Thus, stromal cells play a role in Treg cell induction that may be similar to the role of professional APCs and use a variety of functions to alter/induce intestinal Treg cells.

## Cofactors That Contribute to Mucosal pTreg-Cell Induction

Cyclooxygenase 2 (COX-2) is an enzyme involved in the conversion of arachidonic acid (AA) to prostaglandins (PG). Prostaglandins are typically induced upon inflammation; however, in the SI COX-2 is present during homeostasis and expressed by SI stromal cells and macrophages (94, 95). The predominant COX-2-dependent metabolite of AA in the intestine is PGE_2_, and mice lacking COX-2 have undetectable intestinal PGE_2_ that leads to increased inflammation upon challenge with dextran sulphate sodium (DSS) (96). Moreover, COX-2 inhibition during dietary antigen feeding in mice elecits loss of oral tolerance with pathological changes in the proximal SI including increased epithelial crypt elongation, villus blunting, and increased LP mononuclear cell proliferation (95). These histological features highly resemble the histological changes seen in patients with Celiac disease caused by aberrant oral tolerance to the dietary protein gluten (97). In keeping with these findings, COX-2-derived PGs enhance SI Treg cell induction in the mLN after OVA ingestion *via* suppression of IL-4 production and GATA3 expression (98). This mechanism is dependent on mLN DCs which are high COX-2 expressers and dependent on PGE_2_. Altogether, these studies show that local COX-2 contributes to SI Treg cell induction and subsequent oral tolerance. For an elaborate discussion about the role of COX-2 in IBD and colorectal cancer the reader is referred to a review by Wang and Dubois (99).

Finally, an additional mechanism by which DCs have been shown to induce Treg cells is through indoleamine 2,3-dioxygenase (IDO). IDO is expressed by CD11c^+^CD103^+^ DCs, reduces local tryptophan concentration, and produces tryptophan metabolites to promote a tolerogenic environment, possibly through Treg cell induction *via* aryl hydrocarbon receptor signaling (100–102). Together, these data emphasize that mucosally induced pTreg differentiation during oral tolerance is highly regulated and dependent on numerous local, tissue-specific, interactions.

## Additional Cues From the Microenvironment Influence Intestinal Treg Cells

Factors that regulate FOXP3 expression include TCR signaling, co-stimulation, cytokine-mediated signals, and the epigenetic status of the *FOXP3* locus (103). In the thymus, *Foxp3* expression is induced following three signals: (1) TCR; (2) co-stimulation by CD28; and (3) IL-2R (64). In contrast, the induction of peripheral Treg cells from naïve CD4^+^ T cells is dependent on a TCR signal and the cytokine TGFβ. In the thymus TGFβ-signaling is not required for FOXP3 induction but rather is required for survival of newly generated Treg cells (104, 105).

Studies examining the regulation of *Foxp3* expression showed that the *Foxp3* promotor has several enhancer elements, found in conserved non-coding sequences (CNS) 1 through 3 (106, 107). Histone methylation of these enhancer elements regulates transcription of *Foxp3*, as shown for the first time by Kim and Leonard (107). The CNS regulating *Foxp3* expression each have different functions. TGFβ-dependent induction of pTreg cells relies on CNS1 located within the *Foxp3* locus and deficiency of this region selectively reduces intestinal pTreg cells without affecting tTreg cells (55, 108). Thus, selective targeting of CNS1 enables the study of extrathymically generated Treg cells, with the caveat that CNS3 also contributes to pTreg cell induction, albeit to a lesser extent (55).

Distinguishing tTreg cells from pTreg cells remains challenging. The transcription factor HELIOS was initially described as a marker to discriminate tTreg cells and pTreg cells, where tTreg cells express HELIOS and pTreg cells do not (57). However, there is conflicting evidence and this topic remains controversial (109). Another marker implicated in the distinction between tTreg and pTreg cells is NEUROPILIN-1 (NRP1). Expression of NRP1 on murine Treg cells is controlled by TGFβ both *in vitro* and *in vivo* (58). In the absence of inflammation, NRP1 appears to predominate in tTreg cells and exhibits low expression in pTreg cells (58, 59). However, Treg cells at sites of inflammation can also be NRP1^high^, confounding this distinction (58). Moreover, TCR sequencing of Treg cells isolated from different compartments (thymus, colon, mLN) show that Treg cells cluster according to their location rather than NRP1 expression, indicating that the TCR repertoire is more similar between compartments than between Treg cells with similar NRP1 expression (110). Altogether, the immunophenotype and functional differences of tTreg cells *versus* pTreg cells remain active areas of investigation.

Under homeostatic conditions, Treg cell numbers are generally associated with the function of the intestinal site in which they are found (15). In the SI, the major function is the uptake of soluble, luminal, food antigens. Conceptually it makes sense that the SI microenvironment is well-suited to promote Treg cell induction to promote tolerance to food antigens (74). Tolerance to an antigen is actively acquired and results in local and systemic immune unresponsiveness specific to the ingested antigen. As food deprivation studies are considerably more difficult to perform given their inherent ethical considerations, most of our knowledge about Treg cell induction in the SI is limited to supplemented model dietary antigens. Oral tolerance to OVA is induced in naïve mice upon by direct intragastric administration of OVA or providing OVA in the drinking water. Both local and systemic unresponsiveness are dependent on *de novo* induction of pTreg cells, while tTreg cells are dispensable for oral tolerance to soluble food antigens (111).

In contrast to the SI, Treg cell induction in the colon is largely associated with gut resident bacteria. During the resolution of adoptive transfer colitis with Treg cells, almost all Treg cells in the spleen, mLN, and colon originate from transferred Treg cells. Thus, the majority of cLP Treg cells in the transfer model is not induced from donor naïve T cells but most likely reflects proliferation of transferred Treg cell population in a lymphopenic host, typically referred to as homeostatic proliferation (112). However, these observations do not yield information pertaining to the homeostatic induction of intestinal Treg cells.

Germ-free mice lacking all microbes exhibit an immature mucosal immune system with considerably lower Treg cell frequencies as compared to mice housed under SPF conditions (113–115). Similarly, administering antibiotics to SPF mice reduces the frequency of colonic Treg cells in conventionally housed mice, though neither GF mice nor antibiotic treated SPF mice are entirely devoid of Treg cells (113). In the SI of both GF and antibiotic treated SPF mice, Treg cell frequency and numbers resemble those of conventionally housed mice, supporting the notion the Treg cells in this anatomic location are primarily responsive to dietary antigens (113, 116). In a landmark paper, Atarashi and colleagues showed that colonic Treg cell induction can be directly modulated by *Clostridia* species (113). The authors inoculated GF mice with a cocktail of 46 strains of *Clostridia* or 16 strains of *Bacteroides*, which increases colonic Treg cells as compared to colonization with SPF stool, with the *Clostridia* cocktail yielding the highest level of Treg cell induction (113). Expansion of *Clostridia* species is associated with a TGFβ-rich environment and, in support of microbial antigens driving colonic Treg induction, there is no increase in SI Treg cell frequency following colonization with *Clostridia* species. These findings also hold true for human *Clostridia* isolates, suggesting that these bacteria may also be relevant for colonic Treg cell induction in humans (117). Thus, the intimate relationship between commensal bacteria and the immune system drives colonic Treg-cell induction. Studying changes in the Treg cell population following manipulation of dietary or microbial antigens can elucidate factors that modulate intestinal Treg cells (92).

Similar to mucosally induced pTreg induction to dietary antigen in the SI, pTreg cells can be induced to microbial protein antigens as well. Using a commensal microbe-derived (Cbir) flagellin expressed by a subset of the Clostridium XIVa cluster of bacteria bound to the tolerogenic, non-toxic portion of cholera toxin, it was demonstrated that APCs induce antigen-specific intestinal Treg cells in an endogenous TGFβ−dependent manner (118). This observation of microbial influence on tolerogenic intestinal Treg cells once more emphasizes the critical role of the gut microbiome in intestinal immune homeostasis.

Elegant studies by Sefik et al. show that colon FOXP3^+^ Treg cells have a markedly distinct transcriptome expressing high levels of *Rorc*, the gene encoding RAR-related orphan receptor gamma-t (RORγt), as compared to Treg cells residing in other locations (50). This was surprising at the time given that RORγt is a known marker for IL-17 producing Th17 cells. These RORγt^+^ Treg cells are NRP1^low^HELIOS^low^, indicating that they are likely pTreg cells. Importantly, RORγt^+^ Treg cells are induced by a diverse group of microbes; moreover, this study suggests short-chain fatty acid (SCFA)-dependent mechanisms make only a minor contribution to colonic Treg cell induction. In addition to the mouse intestine, RORγt^+^ Treg cells have been detected in human colon tissue (50). These RORγt^+^ Treg cells differ from Treg cells expressing IL-33R or GATA3, as these two latter populations are HELIOS^+^ whereas most RORγt^+^ Treg cells are HELIOS^neg^ (54, 119). Interestingly, despite being RORγt^+^, the gene signature of this Treg cell population only partially overlaps with Th17 cells (50). These studies show that Treg cells with different properties are present throughout the intestine.

## Mechanisms of Intestinal Treg Cell Induction Mediated by Intestinal Microbial Metabolites

SCFA are a fermentation byproduct of fiber digestion, which primarily occurs in the colon. Consequently, the concentrations of SCFA are highest in this part of the intestine (120). As expected, GF mice exhibit lower concentrations of SCFA in the intestine, and supplementation of SCFA in the drinking water elevates intestinal SCFA and increases colonic Treg cells (121, 122). In addition to the gut lumen, SCFA are also present in the vena porta blood. The concentration of SCFA in the vena porta is higher than in peripheral blood, indicating that SCFA are transported from the intestine to the liver and from there are taken up and dispersed throughout the periphery (120, 123). SCFA are often considered only present in the colon and not in the SI, but this is an oversimplification. Although concentrations of SCFA are higher in the colon, SCFA are indeed present and absorbed in mouse and human SI (124–126). Since luminal content (e.g., fiber) resides in the SI for a shorter duration than in the colon, SCFA-producing bacteria in the SI have to be more efficient (126).

The three SCFA produced in the gut and shown to be relevant for Treg cell induction are propionate, butyrate, and acetate. Interestingly, these SCFA each have different mechanisms of action. Mechanistically, SCFA signal through three G-protein-coupled receptors (GPCRs): GPR41, GPR43, and GPR109A, and inhibit histone deacetylase (HDAC) (127, 128). HDAC inhibitors increase Treg cell production and promote their suppressive function (129). These GPCRs are expressed by both immune and epithelial cells and their function is diverse (128, 130). GRP109A is only activated by butyrate, but not by acetate or propionate, and GPR109A deficiency was shown to specifically reduce colonic Treg cells (131, 132). Butyrate binding to GPR109A on DCs and macrophages also induces anti-inflammatory molecules, including IL-10 and RALDH to promote pTreg induction in a CNS1-dependent manner and can act directly on T cells to induce FOXP3 by inhibiting HDAC (132, 133). Conversely, all three SCFA are able to activate GPR43. Intestinal Treg cells express relatively high amounts of GPR43, but only in the presence of microbiota. The mechanism by which propionate increases intestinal Treg cell accumulation, by both increasing induction/homing as well as proliferation, likely occurs through GPR43. Smith et al. demonstrated treatment of wild-type mice with propionate increases colonic Treg cell numbers as compared to GPR43-deficient mice also treated with propionate (122). The authors also demonstrate that propionate increases histone acetylation, which was not observed in GPR43-deficient mice. Furthermore, when *Rag* ^-/-^ mice co-injected with naïve T cells and GPR43-sufficient or -deficient Treg cells are treated with propionate, mice receiving GPR43-sufficient Treg cells exhibited greater protection from intestinal inflammation as compared to mice co-injected with GPR43-deficient Treg cells. Not only does GPR43 induce colonic Treg cells, it also promotes proliferation of Treg cells already present. In contrast to propionate, butyrate only increases pTreg-cell induction (not proliferation), whereas acetate does not drive induction but rather Treg cell accumulation, independent of CNS1 (133, 134). The mechanism by which butyrate induces Treg cells is through direct acetylation of the *Foxp3* locus (133). Finally, butyrate and propionate have also been shown to prime DCs to drive Treg cell induction *in vitro* (133). To summarize, SCFA induce intestinal Treg cells through various mechanisms.

Given the abundance of SCFA along the GI tract as discussed above, SCFA are surprisingly important for maintenance of oral tolerance. As compared to mice given a low-fiber diet, the CD11c^+^CD103^+^ DCs in mice fed a high fiber diet display more RALDH activity and induce more antigen-specific Treg cells *in vitro* and *in vivo* (135). *In vivo*, the increase in Treg cells was observed both in mLN as well in the SI LP. By using a vitamin A-deficient diet, the authors showed that Treg cells might also be induced by the high-fiber diet in a CD103^+^-independent manner. However, Treg cells induced in the absence of vitamin A have impaired suppressive capacity, indicating that both fiber and vitamin A are essential for induced Treg cell function in the SI. Lastly, the authors showed that epithelial GRP43 and immune cell GPR109A are essential for induction of tolerance.

Colonic Treg cells can also be induced independent of bacterial-derived SCFA. One reported mechanism that involves the symbiont *Bacteroides fragilis* that produces polysaccharide A (PSA), which induces IL-10 producing FOXP3^+^ Treg cells in a toll-like receptor 2 and plasmacytoid DC-dependent manner (136, 137). PSA does not appear to alter the frequency of colonic Treg cells, but rather enhances their function. Another recently discovered mechanism of colonic Treg induction is through bile acids and bacteria-derived bile acid derivatives. These have been shown to selectively induce colonic RORγt^+^ Treg cells, once more emphasizing the importance of interactions between the gut microbiome and immune system for intestinal Treg cells (138).

These observations show that although there are distinct and similar drivers of Treg cell induction in the SI and colon, the local microenvironment governs the dominant mechanism of Treg cell induction. Whereas Treg cell induction in the SI is driven by DC-presented food antigens in the presence of TGFβ and diet-derived RA, Treg cell induction in the colon largely occurs TCR stimulation in the presence of fermentation products, microbial metabolites, and TGFβ, the latter resulting in tolerance to the resident commensal microbes populating the colon.

## Trafficking of Treg Cells to the Intestine Under Homeostatic Conditions

Migration of conventional T cells occurs *via* expression of adhesion molecules and chemokine receptors and is essential for a physiological immune response (139, 140). T cells migrate to non-lymphoid tissue in the absence of inflammation, and also in response to stimuli such as commensal bacteria and dietary antigens (116, 141, 142). To reach the intestine, Treg cells express homing receptors that partially overlap with those of conventional T cells (143, 144). Lee et al. elegantly showed that tTreg cells first undergo changes in homing receptors to egress the thymus, and migrate to secondary lymphoid tissue where they undergo changes following antigen priming and migration to non-lymphoid tissue (143). GALT and mLN, but not splenic, DCs can induce α_4_β_7_ expression on Treg cells, which enables MADCAM-1-dependent extravasation from circulation into the intestine (144–146). Additionally, they induce expression of the chemokine receptor CCR9, enabling trafficking to the SI (146, 147). Interestingly, *in vivo* induction of these molecules is preferential after oral administration of OVA as compared to i.p. administration, which is attributed to the transport of OVA by DCs to the mLN (145). Induction of α4β7 on Treg cells is more efficient by mLN DCs as compared to DCs from peripheral lymph nodes or spleen and is also attributed to RA (74, 144, 148). These findings can be translated from mice to human as Bakdash et al. showed that human monocyte-derived DCs cultured in presence of RA resemble LP DCs in terms of CD103 expression and RA production (149). When cultured together with T cells, these DCs induce a suppressive FOXP3^neg^ T cell population, as well an IL-10^+^ T cell population, although suppression of naïve T-cell proliferation *in vitro* was independent of IL-10. The RA-conditioned human DCs also induced CCR9 and α4β7 on T cells. Moreover, IL-10 production was only observed in the CCR9^+^ T cells and not in the CCR9^neg^ T cells. In contrast to RA, TGFβ induces FOXP3^+^ cells rather than IL-10 producing T cells in these assays. This human *in vitro* data shows RA induces CCR9 and α4β7 on an IL-10 producing suppressive T cell population with improved intestinal homing capacity, in contrast to TGFβ, which induces FOXP3 expressing T cells. Altogether, these studies demonstrate that RA from CD103^+^ DCs induces CCR9 and α4β7 on Treg cells, which licenses them with improved homing to the SI. In the context of antigen presentation in the lymph nodes, it makes sense that DCs license activated Treg cells to migrate to the site of antigen exposure.

The induction of chemokine receptors in Treg cells can also be independent of RA production by DCs (150). Treg cells can specifically acquire capacity to home to the colon. For instance, the microbiome can modulate expression of GPR15 (150) and GPR15-deficient mice exhibit fewer FOXP3^+^ Treg cells in the colon but not the SI. In addition to GPR15 and α_4_β_7_, colonic Treg cell homing, and homing of other leukocyte subsets, is also dependent on CCR6 (151, 152). Together with the observation that OVA-specific T cells preferentially differentiate into CCR6^+^FOXP3^+^ cells (151) in mucosal tissue upon antigen exposure, the induction of CCR6 expression and subsequent intestinal homing and suppression is likely antigen driven. Further insights in Treg cell homing comes from a study by Nakanishi et al., who utilized a photoconvertible mouse, showing for the first time that Treg cells can move bidirectionally between colon and lymph nodes (153). The movement of Treg cells from the colon to the lymph nodes depends largely on Sphingosine-1-phosphate receptor 1, as blocking this pathway with FTY720 (a S1PR1 agonist that downregulates S1PR1), substantially decreases the number of photoconverted cells in the lymph nodes.

In conclusion, Treg cells utilize a variety of trafficking molecules to migrate bidirectionally between lymph nodes and mucosal tissues in an antigen-dependent manner.

## The Sustenance of Intestinal Treg Cells Is a Delicate Balance Between Apoptosis and Proliferation

In contrast to early viewpoints that Treg cells are quiescent, Treg cells are now known to have substantial proliferative capacity. Liston and Gray reviewed the turnover of the central Treg cell population in circulation and in lymphoid organs (35). Nearly half of the circulating Treg cells in both humans and mice undergo division every eight to ten days (154, 155). Under homeostatic conditions, a high rate of proliferation is balanced out by a high rate of apoptosis with the latter occurring in Treg cells through FOXP3-dependent phosphorylation of the pro-apoptotic protein BIM (156). BIM is antagonized *via* MCL1 through IL-2 signaling (154) and under steady-state conditions, Treg cells repress paracrine and autocrine IL-2 production. The high affinity IL-2R is a trimeric protein, consisting of an IL-2Rα (CD25), IL-2Rβ (CD122), and IL-2Rγ (CD132) subunit with Treg cells constitutively expressing CD25. In the absence of CD25, the IL-2Rβ and IL-2Rγ exhibit intermediate-affinity for IL-2 (157). Thus, Treg cells exhibit increased sensitivity to IL-2 as compared to conventional T cells.

Following partial depletion of Treg cells, apoptosis decreases, and proliferation increases in an IL-2-dependent manner, likely through the “de-repression” of IL-2 production. This balance of proliferation and apoptosis of central Treg cells is thought to drive a homeostatic network of rapid self-correction and IL-2-dependent competition effects (35). In the SI, the IL-2 for intestinal Treg cells is in part supplied by group 3 innate lymphoid cells (ILC3s), whereas in the cLP conventional T cells are a significant source of IL-2. Furthermore, the lifespan of Treg cells also differs in various compartments. In contrast to the half-life of circulatory Treg cells, the half-life of tissue resident Treg cells in the intestine appears longer. For example, the estimated half-life of pTreg cells in the SI LP has been is 4-6 weeks, which is based on the sharp decline of SI Treg cells in mice after deprivation of Treg inducing food antigens (51).

The mechanisms that maintain Treg cells in non-lymphoid tissues differs from central Treg cells. Rather, the transcription factor BLIMP-1 (encoded by *Prdm1*) is expressed by effector Treg cells and together with IRF4, is required for IL-10 production (45). In T cells, BLIMP-1 is induced by IL-2 and has a negative feedback loop for IL-2 production (158, 159). In BLIMP-1-deficient mice, activated T cells accumulate in the liver, lung and intestine, leading to inflammation in these organs (160, 161). Treg cells express high levels of BLIMP-1, suggesting that the autoimmune pathology in BLIMP-1-deficient mice may be due to defects in Treg cells (160, 161). BLIMP-1^+^ Treg cells produce IL-10 and resemble an effector Treg cell phenotype, whereas IL-10 is not produced by BLIMP-1-deficient Treg cells (45). In Treg cells specifically, BLIMP-1 represses the pro-survival protein BCL-2, and the chemokine receptor CCR6 that mediates trafficking of T cells to the intestine. Thus, BLIMP-1 may antagonize the accumulation of effector Treg cells in the intestine (45). The similarities between *Il10* ^-/-^ and BLIMP-1-deficient mice suggests that the pathology in BLIMP-1-deficient mice may be attributed to a lack of IL-10 production by effector Treg cells (45). Altogether these studies identify BLIMP-1 as an important factor in intestinal Treg cell homeostasis, preventing auto-immune disease through modulation of sustenance and function of Treg cells.

Interestingly, modulation of food antigens reduces the frequency of RORγt^neg^ Treg cells in the intestine, whereas manipulation of the microbiome reduces the RORγt^+^ Treg cells (51). Manipulation of the microbiome does not reduce GATA3^+^/Helios^+^ RORγt^neg^ Treg cells, indicating that these Treg cell subsets are not sustained by microbial signals but rather by other factors such as IL-33 (54, 162). Meanwhile, in GF or antibiotic treated mice, the proportion of Treg cells that are IL-10^+^ decreases specifically in the cLP (90). Thus, the gut microbiome is essential to sustain both the intestinal RORγt^+^ Treg and IL-10^+^ cLP Treg cell compartment.

To summarize, IL-2 is a key cytokine involved in Treg cell maintenance and regulates the balance between apoptosis and proliferation *via* redundant mechanisms. The central Treg cell turnover is higher as compared to intestinal Treg cells with intestinal Treg cells having different mechanisms of sustenance.

## Intestinal Treg Cells Reside in Lymphoid Follicles and Are Interspersed Throughout the Lamina Propria

Under homeostatic conditions, Treg cell frequency increases from duodenum to jejunum and from ileum to colon (163). In mice, colonic Treg cells reside in the LP and under homeostatic conditions are mostly observed in lymphoid clusters (112). In a model where mice reconstituted with OVA-specific T cells are challenged with OVA-producing bacteria, cLP Treg cells are preferentially found in lymphoid follicles and colocalize with DCs while macrophages reside outside the follicles (164). The Treg cells actively suppress effector T cell cytokine production, as indicated by lower IFNγ production in stimulated T cells from mice that receive OVA-specific Treg cells (together with conventional T cells) as compared to mice that receive only OVA-specific conventional T cells.

Similar to mice, most Treg cells in the human colon are found in lymphoid follicles, with a smaller fraction scattered throughout the LP (112). Interestingly, decreased intestinal Treg cells have not been observed in patients with IBD, nor do these Treg cells exhibit reduced suppressive capacity *in vitro* as compared to healthy controls (165–170). Rather, during colonic inflammation the number of colonic Treg cells increases, whereas the number of colonic T cells remains rather unchanged (112, 171). Although the localization of Treg cells in IBD is often not detailed, the FOXP3^+^ T cells in patients with IBD appear to colocalize with lymphocytes, which begs the question: why are they not able to suppress colitogenic T cell responses? (172) Several possibilities have been put forward. For example, it has been reported that LP T cells might be resistant to Treg cell mediated suppression in a SMAD7-dependent manner (173). Furthermore, the local inflammatory microenvironment may attenuate Treg cell function during active inflammation, effector Treg cells may transiently upregulate FOXP3, or the FOXP3^+^ cells represent an uncommitted, i.e. non-suppressive, population of FOXP3^+^ cells that proliferate within an inflammatory micro-environment (174, 175). Recent work using single-cell RNA-seq uncovered marked changed in T cell expression profiles in patients with ileal Crohn’s disease depending on whether the cells are from inflammatory lesions or adjacent non-inflamed tissue (176).

In contrast to the LP and its lymphoid follicles, murine Treg cells are sparse in the intestinal epithelium. Rather, amongst lineage labelled Treg cells that have migrated into the epithelium, 50% and 10% of the SI and cLP Treg cells, respectively, lose FOXP3 expression (177). Interestingly, when mice are treated with broad-spectrum antibiotics the number of Treg cells in the SI epithelium increases. Thus, this study shows that in the SI, the microbiome destabilizes epithelial Treg cells.

## Intestinal Treg Cell Maintain Intestinal Immune Homeostasis *via* Contact-Dependent and -Independent Mechanisms

Most evidence for the role of intestinal Treg cells in maintaining intestinal immune homeostasis comes from studies showing dysfunction of Treg cells in mice. An often-used model is the adoptive T cell transfer model of colitis. In this model, immunodeficient mice lacking mature T and B cells (e.g., *Rag1 ^-/-^*) develop colitis a few weeks following the transfer of naïve CD4^+^ T cells (CD25^neg^CD45RB^high^) unless a fraction containing Treg cells (e.g., CD45RB^low^ or CD4^+^CD25^+^FOXP3^+^) are co-injected (10, 178). The intestinal inflammation in the adoptive T cell transfer model of colitis is mediated by inflammatory cytokines produced by effector T cells in response to gut microbial antigens and requires the presence of *Helicobacter* spp (179–182). Although inflammation is generally restricted to the large intestine, there can be limited small bowel involvement as well (178). The adoptive T cell transfer colitis model illustrates how Treg cells are instrumental in controlling effector T cell responses to prevent T cell-mediated inflammation in the gut. Thus, immune homeostasis in the intestine requires tolerance to commensal intestinal microbiota.

The adoptive transfer model of colitis really allows to tease apart Treg cell function. For example, Denning et al. performed adoptive transfer experiments using CD4^+^CD25^+^β7^-/-^ Treg cells and found that β7^-/-^ Treg cells are impaired in intestinal homing, but still have comparable suppressive function as wild-type Treg cells *in vitro* as well as *in vivo* (183). Thus, migration and suppressive function of Treg cells may be independent processes.

The presence of tTreg cells without intestinal pTreg cells is insufficient to maintain intestinal immune homeostasis, as CNS1-deficient mice develop Th2 skewed intestinal inflammation (55). The Th2-type intestinal inflammation, rather than Th1 or Th17, is attributed to GATA3-specific Treg cell function (55). Evidence for synergistic roles between pTreg cells and tTreg cells further comes from studies examining the treatment of established adoptive transfer colitis (12) with Treg cells. Haribhai et al. generated *in situ* induced Treg cells (pTreg cells) by transferring CD45RB^high^ naïve T cells into *Rag1^-/-^*mice and found that these pTreg cells exhibit comparable suppressive function *in vitro* as compared to *ex vivo* Treg cells isolated from the spleen and mLN (184). Induced mLN Treg cells have a slightly different phenotype than mLN Treg cells from a wildtype mouse, in terms of CD25 (higher during inflammation), CD62 (lower during inflammation), and CD103 (lower during inflammation) expression. This finding supports that the pTreg cells are indeed suppressive and also shows that pTreg cells in the mLN during inflammation are dissimilar from Treg cells residing in the mLN under homeostatic conditions. The authors then switched to showing the function of pTreg cells during intestinal inflammation but first examined the function of *in vitro* induced Treg cells (iTreg cells) with *ex vivo* Treg cells. They used the iTreg cells as a model for *in situ* pTreg cells – it is not feasible to get enough *in situ* pTreg Treg cells from the cLP in this model to treat other mice with. Mice transferred with *ex vivo* Treg cells had significantly less colitis as compared to mice transferred with iTreg cells, which shows that iTreg cells have dissimilar suppressive capacity *in vitro* as compared to *ex vivo* Treg cells, which, as we now know and as discussed above, are a group of both pTreg cells and tTreg cells. Subsequently, the rescue of adoptive transfer colitis was examined in absence of pTreg cells by transferring in naïve T cells from mice lacking functional FOXP3 (*Foxp3* ^ΔEGFP^). Interestingly, a combination of *ex vivo* Treg cells and iTreg cells ameliorated established disease whereas *ex vivo* Treg cells alone or iTreg cells could not. Altogether, this study shows that resolution of intestinal inflammation it is beneficial to have a combination of *in vitro* induced Treg cells and *ex vivo* Treg cells.

**Figure 2:**
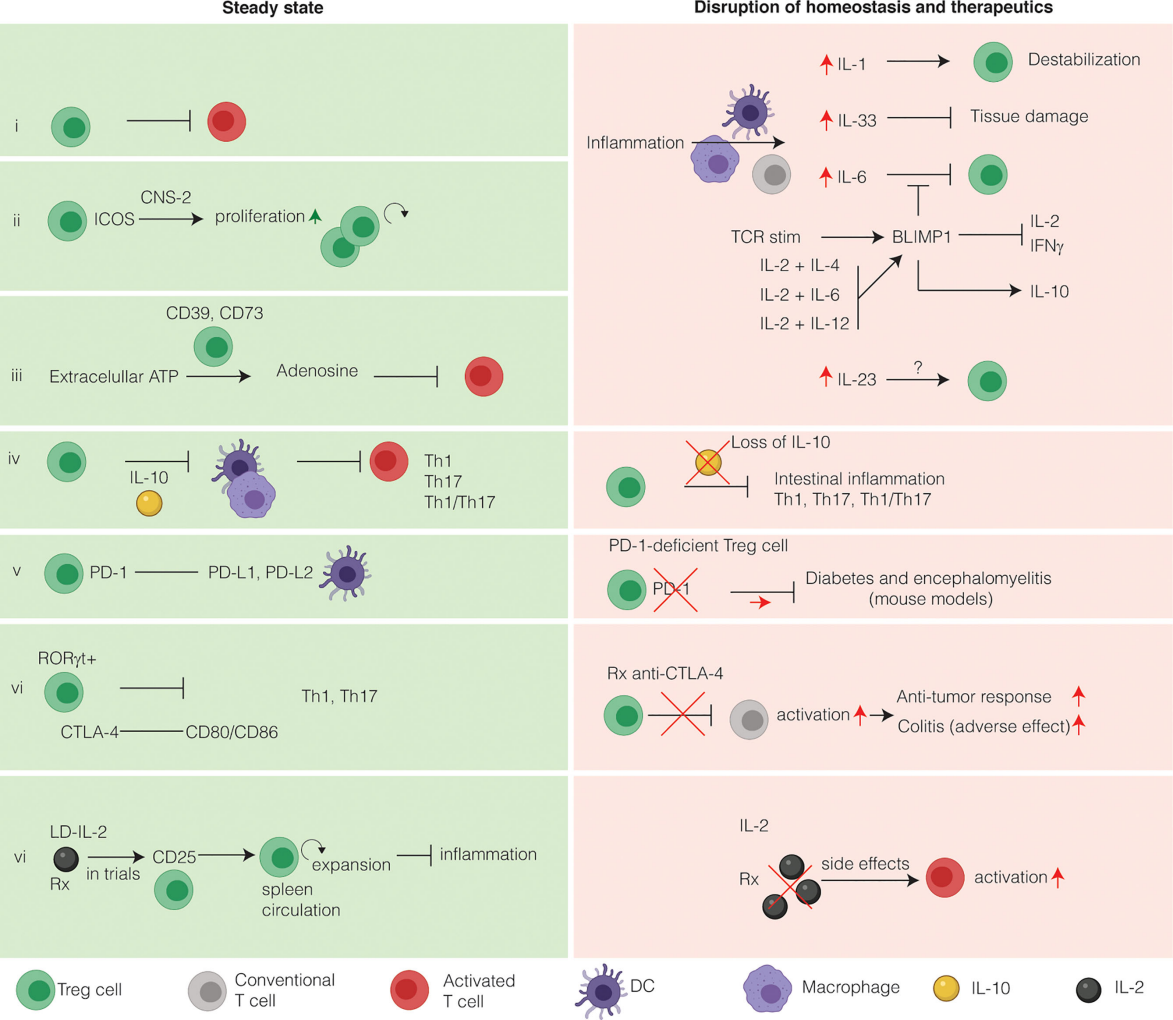
**Intestinal Treg cells tightly maintain intestinal immune homeostasis and relevance for immunotherapy**. Intestinal Treg cells have several markers that discriminate them from other Treg cells. Under homeostatic conditions, Treg cells suppress effector T cells to prevent inflammation (i). During inflammation, several inflammatory cytokines alter the phenotype of intestinal Treg cells. (ii) Inducible co-stimulator (ICOS) stabilizes intestinal Treg cells in a CNS2-dependent manner. (iii) Extracellular ATP is converted to the immunosuppressive adenosine by Treg cells expressing CD39 and CD73. CD73 is induced by TGFβ. (iv) The main immunosuppressive cytokine secreted by intestinal Treg cells is IL-10. IL-10 functions to inhibit T-helper 1 (Th1), Th17 and Th1/Th17 inflammation in a STAT3-dependent manner *via* IL-10R signaling on APCs, limiting inflammasome activation. On the other hand, loss of IL-10 both in mice and in humans leads to intestinal inflammation and IBD, respectively. The main co-stimulatory receptors expressed on Treg cells are programmed death receptor (PD-1) (v) and cytotoxic T-lymphocyte associated protein 4 (CTLA-4) (vi). Both these co-stimulatory receptors are exploited therapeutically to increase the anti-cancer immune response. Anti-CTLA-4 therapy has colitis as a frequent side-effect, whereas colitis is not associated with blockade of the PD-1 pathway. (vi) Because Treg cells express the high affinity receptor CD25, they respond to low doses of IL-2 by expansion, thus limiting inflammation. LD-IL-2 is investigated in a clinical trial for its use in patients with IBD. In contract to LD-IL-2, a high dose of IL-2 will activate T cells and lead to deleterious side effects and therefore is not used in clinical practice.

Treg cells can exert their suppressive effects using different modes of action (**Figure 2**). These suppressive mechanisms include production of tolerogenic cytokines, cytolysis, metabolic disruption, and the modulation of APC function (185). Colonic Treg cells have a transcriptome that resembles highly suppressive Treg cells (50). Recently, a group performed single-cell RNA-seq on Treg cells and memory T cells isolated from the colon, skin, and respective draining lymph nodes under homeostatic conditions (40). Treg cells from the colon differ from Treg cells in the draining lymph nodes by differential gene expression related to the TNFR-NF-κB pathway, and several functional genes, e.g. *Il10*, *granzyme b* (*Gzmb)*, and *cytotoxic lymphocyte antigen 4* (*Ctla4)*. In the lymphoid tissue specifically, a small population of Treg cells expresses genes overlapping with effector Treg cells and non-lymphoid tissue T cells, suggesting functional migration to non-lymphoid tissue. In the absence of intestinal inflammation, colon Treg cells travelling to lymph nodes express higher levels of immunosuppressive molecules (ICOS and LAG3) than lymph node Treg cells that have not trafficked to the colon (153). The same observation was made during DSS-induced intestinal inflammation. That is, expression of ICOS, PD-1, CTLA-4, and IL-10 is higher in cLP Treg cells then in lymph node Treg cells. An *in vitro* suppression assay showed that colonic Treg cells are superior suppressors of T-cell proliferation as compared to lymph node Treg cells, suggesting that highly suppressive Treg cells migrate back to the lymph nodes during inflammation.

The suppressive phenotype of intestinal Treg cells is related to their regulatory function. CTLA-4 functions as a co-inhibitory molecule on T cells by binding CD80/CD86 on activated APCs (186), is critical for Treg-cell function to prevent lethal auto-immunity in mice, and is highly expressed by intestinal Rorγt^+^ Treg cells (50, 162, 187). This is also true in humans as patients with CTLA-4 haploinsufficiency have impaired Treg cell function and are highly susceptible to very-early-onset Crohn’s-like intestinal inflammation (188–190). In agreement with this, colitis is one of the most common side-effects of immune checkpoint blockade with anti-CTLA-4 in cancer (191).

Another co-inhibitory receptor expressed on Treg cells is programmed death receptor 1 (PD-1), although the role of this receptor has not been fully dissected (192). PD-1 functions by binding to PD ligand 1 (PD-L1) and PD-L2 and signaling *via* this receptor-ligand complex results in inhibition of CD28, thus inhibiting co-stimulation of T cells in the early phase after antigen encounter (193). PD-L1 synergizes with TGF-β to promote pTreg cell induction *via* PD-1 on differentiating Treg cells (192, 194, 195). Moreover, several studies argue that PD-1 is involved in Treg cell maintenance through maintaining the suppressive phenotype (196), preventing conversion of Treg cells into pro-inflammatory effector memory T cells (197) and rendering them less sensitive to apoptosis (198). Initially, PD-1 signaling was suggested to may have less profound effects on suppressive function as IL-2-induced Treg cells isolated from PD-1 deficient mice are not altered in their suppressive capacity (198). However, the recent generation of mice that selectively lack PD-1 in Treg cells allowed to establish that PD-1 deficient Treg have an activated phenotype and exert enhanced immunosuppressive function, as compared to wild-type Treg cells, in experimental diabetes and autoimmune encephalomyelitis (199). These studies would argue that blockade of PD-1 on Treg cells should enhance their regulatory capacity rather than diminish it. In keeping with this hypothesis the prevalence of colitis as adverse effect in cancer patients receiving PD-1 blockade is much lower than in patients receiving anti-CTLA-4 therapy (200). Of note, the role of PD-1 in Treg cells in IBD has mostly focused on IL-10 producing Tr1 cells (201). PD-1 expression on intestinal CD4^+^Foxp3^neg^ Tr1 cells enriches for IL-10 secreting cells. In mice, co-transfer of IL-10-producing CCR5^+^PD-1^+^ Tr1 cells strongly inhibits colitis induced by transfer of Th17 cells, whereas IL-10-producing control T cells lacking CCR5 and PD-1 are less efficient (202).

Besides the enrichment in co-inhibitory receptors Treg cells also carry co-stimulatory receptors. In particular, inducible co-stimulator (ICOS) is involved in Treg cell maintenance. ICOS-deficient mice have reduced frequencies of Treg cells in secondary lymphoid tissues but do not develop spontaneous disease when housed under SPF conditions (203). In mice with specific deletion of ICOS in Treg cells, thymic output of Treg cells and the numbers of intestinal IL-10^+^ cells are increased, perhaps as a compensatory mechanism (203). However, together with the fact that ICOS-deficient Treg cells are equally suppressive as wildtype Treg cells in adoptive transfer colitis it appears that ICOS plays a key role in the stability of intestinal pTreg cells rather than directly regulating suppressive capacity. In line with this, ICOS increases antigen-specific Treg cell expansion in a CNS2-dependent manner (204). In the intestinal LP ICOS-L is expressed by CD11c^+^CD103^+^ DCs, suggesting these APCs can provide a signal to sustain the intestinal ICOS^+^ Treg cell population (203). This ICOS mediated sustenance is important as, although ICOS-deficient Treg cells are equally suppressive as wildtype Treg cells in adoptive transfer colitis they eventually cannot prevent mortality in Foxp3 deficient mice (203, 204). Moreover, ICOS-deficient Treg cells cannot reverse established adoptive transfer colitis (203). Altogether, these results indicate that ICOS is required for the stability of intestinal Treg cells to maintain suppressive function during intestinal inflammation.

Treg cells also exert suppressive function by metabolic disruption. Treg cells express both CD39 and CD73 (205, 206), and SI Treg cells in particular express high levels of CD39 and CD73 in comparison to Treg cells from other compartments (207). The expression of CD73, but not CD39, is regulated by TGFβ yet both CD39 and CD73 convert pro-inflammatory extracellular ATP into immunosuppressive adenosine (208, 209). In T cells, adenosine signals *via* the A2A adenosine receptor and is required for the suppression of intestinal inflammation independent of TGFβ and IL-10 (210–212). However, a surplus of adenosine in adenosine deaminase deficiency results in severe combined immunodeficiency (SCID) [for the function of Treg cells in relation to autoimmunity in ADA-SCID see Sauer et al. (213)]. Altogether, these studies identify a role for metabolic disruption of intestinal Treg cells during intestinal inflammation.

A cell-contact dependent mechanism intestinal Treg cells employ is through latent activation gene 3 (LAG-3) (214). The mechanism of LAG-3 mediated suppression occurs *via* inhibition of production of the inflammatory cytokine IL-23 by CX3CR1^+^ macrophages, which promotes IL-22 production by group 3 innate lymphoid cells (ILC3s) during anti-CD40 colitis (215). Another mechanism Treg cells may use to suppress effector T cells is GZMB that functions to lyse target cells (e.g., effector T cells) but to date the role for GZMB in intestinal Treg cells is lacking.

Intestinal Treg cells also maintain intestinal immune homeostasis through secretion of immunosuppressive cytokines including IL-10, TGFβ, and IL-35. The complex role of TGFβ in Treg cells has been extensively reviewed by Sakaguchi et al. (9). The importance of IL-10 and the intestinal microbiome as a driver of inflammation was initially described by Kuhn and colleagues who discovered spontaneous enterocolitis development in IL-10-deficient mice housed under non-specific-pathogen free (SPF) conditions (216). Later studies showed that colitis in SPF housed *Il10* ^-/-^ mice required the presence of *Helicobacter species* (217). Although IL-10 can be secreted by multiple types of immune cells, IL-10 produced by FOXP3^+^ Treg cells plays an especially critical role in maintaining colonic immune homeostasis (218). IL-10 production by FOXP3^+^ Treg cells is found more frequently in the colon as compared to SI and secondary lymphoid tissue and is regulated by the microbiome (113, 219). In humans, deficiency in IL-10 production or IL-10 receptor signaling causes infantile colitis (220–222). The IL-10 receptor is more abundantly expressed on APCs than T cells (223), and cell-specific deletion of the IL-10R alpha chain in APCs is sufficient to induce colitis (44, 224, 225). IL-10R signaling activates the transcription factor STAT3 and perhaps not surprising, global or DC-specific deficiency for STAT3 largely phenocopies the pathology of *Il10* ^-/-^ mice (36, 226). In macrophages, autocrine IL-10 signaling impairs inflammasome activation (227). *Via* caspase, activation of the inflammasome results in increased levels of active IL-1β (228). Fitting to this pathway, inhibition of IL-1β with anakinra in IL-10R deficient patients decreases intestinal inflammation (229, 230). The consequence of IL-10-mediated regulation of intestinal APCs is largely though inhibition of inflammatory Th17 (CD4^+^IL-17A^+^) cells, Th1 (CD4^+^IFNγ^+^) cells (42, 43) as well as IL-17A^+^IFNγ^+^CD4^+^ double positive T cells (47). In summary, IL-10 is a central, non-redundant, effector cytokine used by intestinal Treg cells to suppress inflammation in the murine GI-tract.

The versatile role of intestinal Treg cells during inflammation is exemplified by a study by Schiering et al., who investigated the role of tissue specificity of Treg cells in a setting of inflammation (54). They found that signaling in colonic Treg cells by the alarmin IL-33 may limit tissue damage, and that the balance between IL-23 and IL-33 might determine the outcome of an inflammatory response in the intestine. Consistent with this, expression of IL-33R (ST2) was found to be increased on colonic Treg cells (54). ST2 expression is generally limited to GATA3^+^ Treg cells and IL-33 increases TGFβ-dependent Treg induction *in vitro* (54). In addition, IL-33 signaling exerts positive feedback on the expression of IL-33R *in vitro (* 54) and increases intestinal Treg cell accumulation, proliferation, and stability under inflammatory conditions by directly binding to the *Foxp3* promotor (54). In an IL-23-dependent model of intestinal inflammation, soluble ST2 was found to be released by stromal cells (54) and has been suggested to act as a decoy receptor to antagonize IL-33 activity. Finally, IL-33 also acts on epithelial cells to produce retinoic acid and thymic stromal lymphopoietin, independent of DCs (231). Together, these studies indicate that the IL-33 pathway regulates intestinal Treg cells to yield a tissue protective response.

Another key cytokine driving IBD pathogenesis is IL-23. IL-23 is a pro-inflammatory cytokine maintaining the stability of Th17 cells. To date, surprisingly little is known about the cell-specific contribution of IL-23R signaling in driving inflammation. It has been reported that *Il23r* is highly expressed on RORγt^+^ colonic Treg cells (50). Moreover, IL-23 may inhibit intestinal Treg cell induction while other studies have failed to show a role of IL-23R signaling in Treg cells (46, 232). *In vitro* Treg-cell induction studies have demonstrated that IL-23 impairs the responsiveness of Treg cells to IL-33 (54). Furthermore, ST2-deficient T cells were impaired in pTreg induction as compared to wild-type T cells in a *Il23a* ^-/-^ host. Finally, ST2^+^ Treg cells from the colon express *Il23r* and IL-33 signaling induces genes co-regulated by FOXP3 and GATA3, which is inhibited by IL-23 in a STAT3-dependent manner (54). Thus, IL-33 functions to increase intestinal TGFβ pTreg induction and IL-23 inhibits IL-33 action on Treg cells that may contribute to intestinal inflammation. These studies indicate that cytokines present during inflammation modulate intestinal Treg cells in various, yet relatively unexplored, ways.

## Intestinal Treg Cells in Inflammatory Bowel Disease

IBD is a group of chronic inflammatory diseases of the intestine whose incidence is increasing in both the Westernized world and in developing countries (233). Ulcerative colitis (UC) and Crohn’s disease (CD), the most common clinical entities of IBD, are chronic conditions lacking a permanent cure resulting in significant long-term morbidity. Although the exact etiology remains unknown, IBD primarily results from dysregulated immune responses to commensal gut microbiota in genetically predisposed hosts that might be triggered by unknown environmental factors (234). In absence of inflammation, Treg cells mediate tolerance though the secretion of soluble mediators such as IL-10, as well as through direct interaction with other immune cells (9). In the case of IBD, the local microenvironment and the microbiome greatly influences Treg cell function and specificity (235). To date, there is no conclusive evidence that polygenic IBD arises due to numerical insufficiency of Treg cells or FOXP3 dysfunction. Nevertheless, there is ample evidence that functional changes in T cells in polygenic IBD can more severely impact Treg cells (e.g., deficiency in CTLA-4 or IL-10).

In patients with IBD, tolerance to commensal bacteria appears to be compromised, as circulating IgG antibodies to Cbir flagellin are associated with Crohn’s disease (236). Murine models have been employed to address whether such loss of tolerance could occur upon acute gastrointestinal infection eliciting transient bacterial translocation. Indeed, during a gastrointestinal infection with *Toxoplasmosis gondii*, tolerance to commensal bacteria is lost and microbiota-specific Cbir1 TCR-transgenic T cells, which are normally quiescent, become activated and differentiate to inflammatory effector and memory T cells (237, 238). Furthermore, in mice infected with *Toxoplasmosis gondii* Cbir-specific T cells upregulate T-BET and mostly secrete IFNγ, whereas infected mice treated with DSS results in Cbir-specific T cells upregulating RORγt (237). Together, these findings argue that during infection or acute inflammation transient commensal microbiota translocation elicits distinct commensal-specific pathogenic memory T cell responses.

In humans, many studies investigating Treg cells rely on changes in peripheral blood Treg cell frequencies as an outcome parameter as blood draws are less invasive than obtaining biopsies through endoscopy and can be better quantitated. However, with the evidence discussed so far, it is clear that peripheral blood Treg cells do not necessarily reflect the complex dynamics of intestinal Treg cells. Therefore, we will limit our discussion to studies examining intestinal Treg cells. During inflammation in patients with IBD, the number of Treg cells in the inflamed tissue typically increases (239). Expression of CCR6 is increased in both inflamed colon as well as in Th17 cells (240, 241). Adoptive transfer studies in mice previously have shown that CCR6-deficient Treg cells are defective in homing capacity and as a consequence, their suppressive function impaired exacerbating colitis as compared to wild-type Treg cells (151). This suppressive defect may be due to a decrease in local IL-10 levels as CCR6 is preferentially expressed by IL-10 producing Treg cells.

IL-10 signaling is highly relevant for a subset of patients with IBD. Rare loss-of-function mutations in the IL-10 pathway are sufficient to cause very-early-onset IBD presenting in children less than 6 years of age (222, 242–245). Polymorphisms of *IL-10*, *IL-10RA*, and *IL-10RB* resulting in functional hypomorphs are also associated with adult onset IBD (246, 247). Conflicting data has been reported regarding the relationship between IL-10 and maintenance of FOXP3 cells in mice and it has been suggested the maintenance of intestinal FOXP3^+^ is dependent on IL-10 only in an inflammatory microenvironment (218, 248–250).

## Monogenic Disorders Driven by Treg Cell Dysfunction

Humans with a spontaneous X-linked mutation in *Foxp3* develop a rare X-linked recessive disorder defined by systemic autoimmunity termed immune dysregulation, polyendocrinopathy, enteropathy, X-linked (IPEX) syndrome (251). Patients presenting with IPEX syndrome lack functional Treg cells due to loss of protein expression or function (252, 253). To date, over 70 mutations are associated with IPEX syndrome, with patients having varying degrees of autoimmunity (254). IPEX syndrome typically presents in male infants with a triad of enteropathy, autoimmune endocrinopathy, and dermatitis (255). In the absence of functional Treg cells, the balance of T helper cells is perturbed, resulting in T cell skewing towards Th2, IL-17 producing T cells, and expansion of autoreactive B cells (256–258). For this reason, inflammation in patients with IPEX syndrome is marked by a Th2 skewed immune response with the patients often having food allergies, eosinophilia, eczema, and elevated levels of serum immunoglobulin E (255, 259). The intestinal pathology in patients with IPEX syndrome mostly involves enteritis and it is atypical for patients to develop large bowel inflammation. Patients with IPEX syndrome commonly have antibodies directed against enterocytes targeting a 75 kDa autoimmune enteropathy related antigen and VILLIN (260–262). Treatment options for patients with IPEX syndrome generally consist of immunosuppression to control autoimmunity, nutritional interventions, and/or allogeneic hematopoietic stem cell transplantation, which can be curative but is not without risk (254).

Other primary immunodeficiencies (PID) have also been described that are caused by diminished Treg cell function independent of FOXP3 (263–266). These PID can occur in both males and females and present with IPEX-like symptoms but due to mutations in *IL2RA*, the gene encoding CD25 which is a component of the interleukin (IL)-2 receptor (267–269). These mutations may have differential effects on Treg cells *versus* effector T cells as CD25 deficiency results in defective IL-10 expression in CD4^+^ T cells (267) and lack of IL-10 expression by Treg cells in mice contributes to intestinal inflammation (46). Recently, a microduplication of the *IL2RA* locus associated with very-early-onset IBD which is attributed to increased IL-2 signaling (270). This increased IL-2 signaling potentiates the IFNγ response after TCR activation (270, 271).

## Low-Dose IL-2 Expands Treg Cells

In recent years, the sensitivity of Treg cells for IL-2 has been exploited for therapeutic purposes to treat chronic inflammatory diseases. Initially therapeutic applications employed high-dose IL-2 to promote effector T cell expansion to treat metastatic melanoma but is rarely used clinically due to deleterious side effects. Conversely, low-dose IL-2 (LD IL-2), selectively promotes the generation, expansion, survival, and functional activity of Treg cells (157). LD IL-2 has been shown to be an effective strategy to expand autologous Treg cells to ameliorate various inflammatory diseases in humans (e.g., GvHD, hepatitis C-induced vasculitis, WAS, and SLE) (157, 272–275). To date it is unknown whether LD IL-2 would be an effective therapeutic used to treat IBD. Using a translational humanized mouse model, LD IL-2 expands human Treg cells and is protective against TNBS-induced colitis (276) that is independent of CD4^+^ HLA restriction (277). Of note, expansion of Treg cells in this model was not observed in the intestine but rather in peripheral sites including blood and spleen. A phase 1b/2a clinical trial investigating the use of LD IL-2 to treat moderate to several ulcerative colitis is ongoing (NCT02200445).

## Enteral Graft *Versus* Host Disease

Patients who undergo allogenic hematopoietic stem cell transplantation frequently develop graft *versus* host disease (GvHD). GvHD can be either acute or chronic, and in most cases the gut is affected. A high frequency of circulatory Treg cells primed to home to the intestine is, perhaps not surprisingly, associated with better outcomes. However, knowledge regarding the role intestinal Treg cells play in GvHD is rather limited (278). One study showed that IL-33R expression in intestinal Treg cells improves their function and since RORγt-deficient Treg cells express higher levels of IL-33R, absence of RORγt results in less severe GvHD (250, 279).

## Outstanding Questions

Additional focus on intestinal Treg cells might yield further insights into Treg cell targeted approaches to mitigate inflammation in patients with IBD and/or autoimmunity. The mechanisms underlying microbial specificity of intestinal Treg cells remain to be determined. Moreover, the extent to which SCFA induce new Treg cells from naïve T cells *versus* increase the proliferation of intestinal Treg cells already present has not yet been fully elucidated (20, 280). A definitive marker to discriminate tTreg cells and pTreg cells is needed to get to the origin of the intestinal Treg cells. Related to the origin, the processes ongoing in lymphoid follicles with regards to intestinal Treg cells remain unclear (281). Together the evidence shows that colonic Treg preferentially localize in lymphoid follicles during homeostasis and inflammation, where they colocalize with DCs and alter the migration and phenotype of conventional T cells. It is tempting to speculate that Treg cells in the lymphoid follicles and those in the LP are phenotypically heterogenous. Much remains to be learned about interaction between epithelial cells, for example whether epithelial cells have differential interactions with Treg cells based on location (i.e., SI *versus* large intestine) remains to be determined. More insight in the interaction between intestinal Treg cells and stromal cells both during homeostasis and disease is especially warranted since mesenchymal stromal cells (mesenchymal stem cells) are now actively being investigated for a range of diseases including Crohn’s disease (282, 283). Last, how Treg cell expression profiles are altered in settings of chronic intestinal inflammation remains to be elucidated.

## Conclusions

In summary, intestinal Treg cells are characterized by a unique transcriptome that is regulated in part by cues from location, site of induction, proximity to the gut microbiome, and interaction with other immune and non-immune cells. Thus, intestinal Treg cells cannot be easily categorized into a distinct and limited number of subsets. Rather, we suggest that the complex local microenvironment, specificity, cell-cell interactions, and signaling receptors contribute to the distinct populations and phenotypes that exhibit functional diversity representing a spectrum of regulators of intestinal immune homeostasis.

## Author Contributions

JJ wrote the manuscript with editing by JS and JG, with input of ER. JL summarized Tr1 cells. The authors approved the final version of the manuscript.

## Funding

The work was supported by an Academy Ter Meulen Grant from the Royal Netherlands Academy of Arts and Sciences and the Cultural Foundation Grant from Prince Bernhard Cultural Foundation (to JJ) and by grants from the National Institute of Diabetes and Digestive and Kidney Diseases DK123489 (to JG).

## Conflict of Interest

The authors declare that the research was conducted in the absence of any commercial or financial relationships that could be construed as a potential conflict of interest.

## Publisher’s Note

All claims expressed in this article are solely those of the authors and do not necessarily represent those of their affiliated organizations, or those of the publisher, the editors and the reviewers. Any product that may be evaluated in this article, or claim that may be made by its manufacturer, is not guaranteed or endorsed by the publisher.

## Abbreviations

AA, Arachidonic acid; CD, Crohn’s disease; CNS1, Conserved noncoding sequence 1; CLTA-4, Cytotoxic lymphocyte antigen-4; DC, Dendritic cell; DSS, dextran sulphate sodium; Foxp3, Forkhead box P3; GALT, Gut associated lymphoid tissue; GF, Germ-free; GPCR, G-protein-coupled receptor; GI-tract, Gastrointestinal tract; GvHD, Graft versus Host Disease; GZMB, Granzyme B; IBD, Inflammatory bowel disease; IL, Interleukin; ICOS, Inducible T-cell costimulator; iTreg, *in vitro* induced Treg cell; IPEX, Immune dysregulation, polyendocrinopathy, enteropathy, X-linked; LP, Lamina propria; mLN, Mesenteric lymph node; MHC, Major histocompatibility complex; NRP1, Neuropilin-1; PD-1, Programmed cell death protein 1; PG, Prostaglandin; pTreg, Peripherally derived Treg cell; PID, Primary immunodeficiency; RA, Retinoic acid; Repertoire, TCR repertoire; RORγt, RAR-related orphan receptor gamma-t; SCFA, Short-chain fatty acids; SI, Small intestine; SPF, Specific pathogen-free; SLE, Systemic lupus erythematosus; tTreg, Thymus-derived Treg cell; Th, T helper; TCR, T cell receptor; Tr1, Type 1 regulatory T cells; Treg cell, Regulatory T cell; TSDR, Treg cell specific demethylation region; UC, Ulcerative Colitis; Treg cell, Regulatory T cell; WAS, Wiskott-Aldrich Syndrome.
